# Characterization and nutritional valorization of agricultural waste corncobs from Italian maize landraces through the growth of medicinal mushrooms

**DOI:** 10.1038/s41598-023-48252-9

**Published:** 2023-11-30

**Authors:** G. Castorina, C. Cappa, N. Negrini, F. Criscuoli, M. C. Casiraghi, A. Marti, M. Rollini, G. Consonni, D. Erba

**Affiliations:** 1 DiSAA, Department of Agricultural and Environmental Sciences - Production, Landscape, Agroenergy, Università degli Studi di Milano, Via Celoria 2, 20133 Milan, Italy; 2 DeFENS, Department of Food, Environmental and Nutritional Sciences, Università degli Studi di Milano, Via Celoria 2, 20133 Milan, Italy

**Keywords:** Biotechnology, Microbiology, Plant sciences

## Abstract

The research investigates the potential use of maize cobs (or corncobs) from five genotypes, including the B73 inbred line and four locally cultivated landraces from Northern Italy, as substrate for implementing Solid State fermentation processes with four Medicinal Mushrooms (MMs). The corncobs were characterized based on their proximate composition, lignin, phenolics content (both free and bound), and total antioxidant capacity. Among the MMs tested, *Pleurotus ostreatus* and *Ganoderma annularis* demonstrated the most robust performance. Their growth was parametrized using Image Analysis technique, and chemical composition of culture samples was characterized compared to that of corncobs alone. In all culture samples, the growth of MMs led to a significant reduction (averaging 40%) in the total phenolics contents compared to that measured in corncobs alone. However, the high content of free phenolics in the cobs negatively impacted the growth of *P. ostreatus*. The final MM-corncob matrix exhibited reduced levels of free sugars and starch (≤ 2.2% DW, as a sum) and increased levels of proteins (up to 5.9% DW) and soluble dietary fiber (up to 5.0% DW), with a notable trend toward higher levels of β-glucan compared to corncobs alone. This research paves the way for the use of this matrix as an active ingredient to enhance the nutritional value of food preparations.

## Introduction

Lignocellulose-containing crop waste materials are globally produced in substantial quantities, often posing a significant threat to the environment^1,2^. The cultivation of edible wood-degrading mushrooms presents nearly limitless possibilities and economically viable potential for the valorization of these substrates. Thanks to their biodegradation capability and nutritional value, mushrooms can serve as valuable tool not only in reducing the environmental impact of agri-food wastes but also in transforming them into new resources for producing high-value food items^2^. In particular, the successful cultivation of edible mushrooms on various agro-waste residues, such as wheat, rice, maize, cotton, soybean, sunflower and maize cob (or corncob), has been proposed for the production of carpophores^3^.

Mushrooms are recognized as one of the most diverse groups of biologically adaptive species. Edible mushrooms serve as valuable source of dietary fibers and proteins, containing most of the essential amino acids. Despite their low fat content, they do contain polyunsaturated fatty acids, particularly linoleic acid (18:2 n6), and oleic acid^4,5^. Furthermore, mushrooms exhibit promising nutritional characteristics, serving as source of vitamins and minerals, enhancing satiety, and potentially reducing the reliance on animal-derived foods^6^. Mycotherapy involving medicinal mushrooms (MMs) has recently received increasing interest in Europe for both preventive and therapeutic purposes^7^. These MMs contain bioactive and health-promoting compounds, including polysaccharides, phenolic compounds, and terpenoids, which demonstrate activities such as antitumoral, antioxidant, immunomodulatory, and antibacterial, relevant for human nutrition and health^8–10^. There is growing focus on the use of MMs for the development of dietary supplements, as they can modulate the immune system and act as anti-inflammatory agents through their antioxidants content^11^. Among MMs, *Ganoderma lucidum*, *Grifola frondosa*, *Lentinula edodes,* and *Pleurotus ostreatus* were the most cited mushrooms in scientific articles (Scopus database) and patents (Espacenet database) in 2020^2^.

The utilization of agricultural materials as substrates for mushroom production represents an alternative method for reducing the adverse environmental impact of their disposal. Edible MMs can be cultivated applying Solid State Fermentation (SSFs) biotechnological processes, involving a bed of solid materials, such as grain straw, brans, and other agro-industrial residues including corncobs, where mushrooms are inoculated in an environment without free-flowing liquid but with adequate water to allow mycelial growth^12^. Macronutrients (C, N, P, K and Mg), along with trace elements (Fe, Se, Zn, Mn, Cu), are essential requirements. Factors such as moisture content, the carbon to nitrogen ratio (C/N ratio), culture pH, and temperature must also be carefully considered for optimal mushroom growth^13^. The moisture content largely depends on the organism and the substrate used for cultivation. The maintenance of an appropriate moisture level within the substrate, ideally within the range 50–75%, is crucial; higher moisture levels may reduce substrate porosity, thus limiting oxygen transfer and inhibiting mushrooms growth^14^.

Maize (*Zea mays*) is one of the most important cereal crops cultivated worldwide along with rice (*Oryza sativa*) and wheat (*Triticum aestivum*). According to the International Grain Council Organization (https://www.igc.int/en/markets/marketinfo-sd.aspx), in the 2020/2021 season 1136.2 million tons of maize have been produced worldwide, of which 136.5, 677.2, 297.6 and 43.9 million tons were used for food, feed, industrial and other uses, respectively. Maize cultivation produces, after grain harvest, different types of agricultural residues including husks (8.4%), leaves (18.5%), stalks (57.8%) and cobs (12.2%). Specifically, corncob is a waste residue produced after the maize is harvested and grains removed, either via a machine or manually. The average yield of corncobs was estimated to be about 14% of grain yield, corresponding to about 16% of the total corn stover in a field^15^.

The corncob is characterized by a cone shaped structure, and represents the main axis (rachis) of the mature female inflorescence (ear), to which the kernels are attached^16^. Corncobs possess distinctive features, such as compact tissue, a high cellulose content, and low levels of ash and nitrogen that make them an interesting source of biomass.

During maize harvesting, after mature corn ears are shelled, corncobs are generally discarded or left unused as agricultural residues, causing a waste of resources and an environmental issue^17^. Unlike stover, corncob waste has minimally effects on soil fertility, owing to its low biodegradability, and its removal from crop fields may increase maize yield^18^. Therefore, the possibility of using corncobs for other applications, including feeding, chemical industry, and fuel production, has been largely explored^19,20^. Several studies concerning the possibility of utilizing corncobs for biotechnological processes have been recently reviewed^21^. These relate to the generation of bioenergies (bioethanol, biohydrogen, biodiesel, biobutanol), production of enzymes (cellulases, xylanases, amylases, glucosidases, pectinases), as well as the production of bioproducts, like xylooligosaccharides, glucose, xylose, lactic acid, and pigments.

The adoption of corncob material for reuse could also prove beneficial in small-scale agricultural settings, such as in the traditional cultivation of local maize varieties. In such scenarios, harvesting is often manual, allowing intact corncobs to be readily available for circular economy–based approaches. Local varieties or landraces refer to populations of crop plants that have adapted to specific agroecological conditions. European maize landraces were developed by farmers during the seventeenth century, following the introduction of this plant species from the Americas^22^; they were predominantly replaced in the 1950s with the introduction of highly productive commercial hybrids. Maize landraces, which were not employed in the breeding process, are currently recognized as the most crucial reservoir of genetic and phenotypic biodiversity for this crop^23,24^. In Italy, these landraces are cultivated in small, distinct geographic areas, isolated form other maize populations, and are primarily used as a fundamental component in the production of traditional food items. Previous studies have underscored the nutritional properties of the flour derived from these varieties^25–29^.

The aim of this study was to investigate the potential utilization of corn cultivation waste as a substrate to apply SSF process to promote the growth of edible MMs. Additionally, the research aimed at providing a comprehensive characterization of a cultivation waste residue, namely corncobs, from four local maize varieties currently cultivated in Northern Italy, and at evaluating the efficacy of selected MMS species for targeted growth on corncobs sourced from the different maize landraces. The findings suggest an innovative approach for re-using and valorizing this material as substrate to apply SSF process for the growth of edible MMs, potentially yielding functional properties and enhanced nutritional value. The repurpose of agricultural waste into valuable products aligns within the circular and bioeconomy framework^21^.

Moreover, the identification of an optimal fungus-substrate combination will contribute to the exploration of the potential use of MMs in the production of new food ingredients. To the best of our knowledge, this study represents a unique endeavor, as there is currently no available data pertaining to the potential functional properties of culture samples derived from maize residue-based SSF setups involving MMs.

## Results and discussion

### Maize corncobs characterization

**Figure 1:**
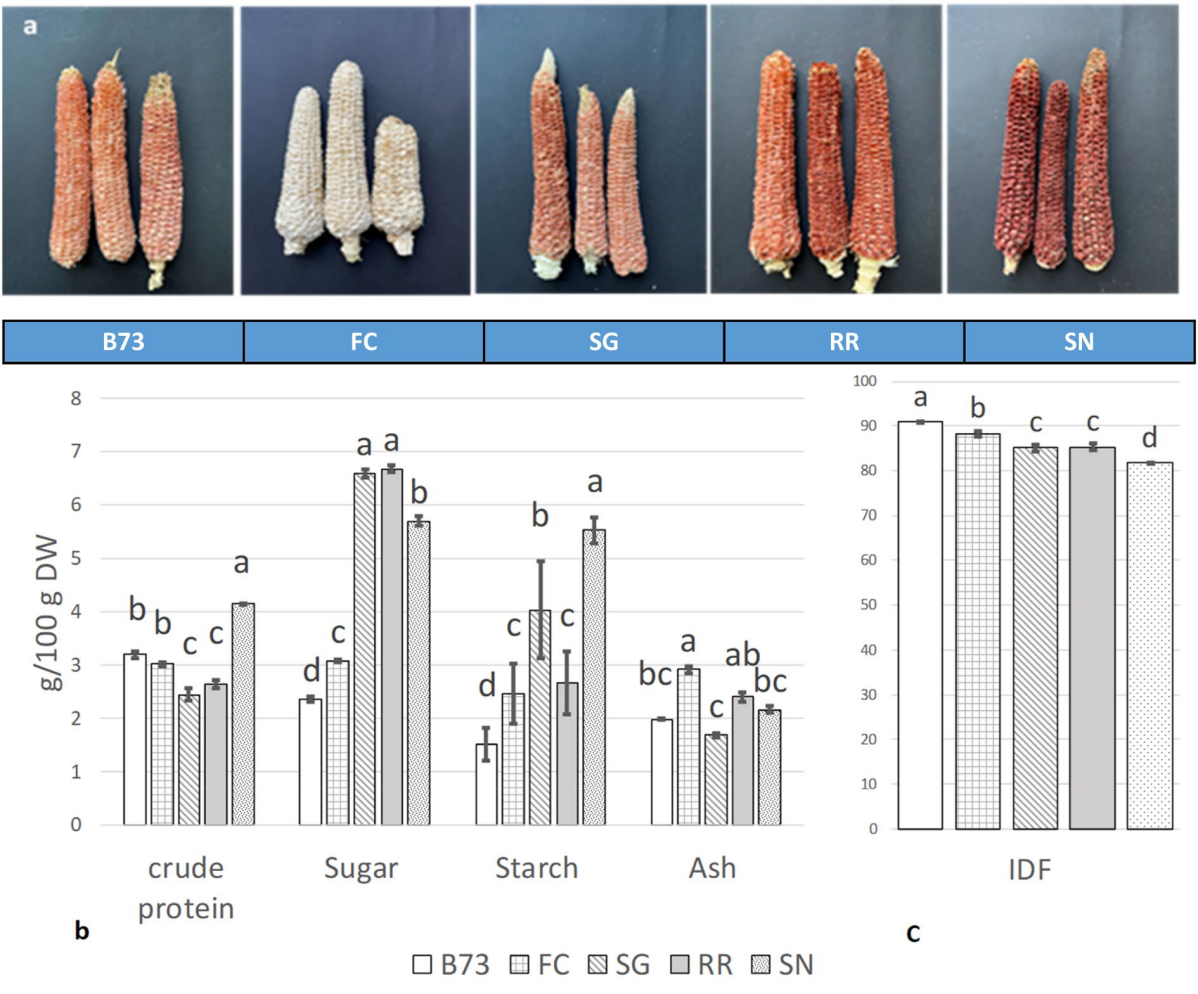
Phenotypes and proximate composition of corncobs. (**a**) Representative images of B73, ‘Fiorine di Clusone’ (FC), ‘Spinato di Gandino’ (SG), ‘Rostrato Rosso di Rovetta’ (RR) and ‘Spinoso Nero Valle Camonica’ (SN) corncobs; (**b**) Contents of crude protein, sugar, starch, ash and (**c**) Insoluble Dietary Fiber (IDF) of the corncobs, expressed as g/100 g DW. Bars represent the means ± SD (n = 3). Within the same variables, bars not sharing common letters are significantly different (*P* < 0.05). IDF: insoluble dietary fiber.

Corncobs derived from the maize inbred line B73 and the local landraces ‘Fiorine di Clusone’ (FC), ‘Spinato di Gandino’ (SG), ‘Rostrato Rosso di Rovetta’ (RR), and ‘Spinoso Nero della Valle Camonica’ (SN) exhibited distinct glume pigmentation patterns (Fig. 1a). The glumes appeared nearly white in FC, while those of B73 and SG displayed a light red/purple hue, and those of RR and SN exhibited a more intense pigmentation. The total monomeric anthocyanins content, estimated after powdering the entire corncob structure and expressed as mg of cyanidin-3-glucoside (the most prevalent anthocyanin in nature) equivalents per 100 g dry weight (mg C3GE/100 g DW), was 3.6 ± 0.43 in B73, FC and SG, 5.0 ± 0.7 in RR, and 15.4 ± 0.3 in SN. These findings seemed consistent with the observed corncob pigmentation across the different genotypes, indicating an increase in color intensity from the light red of B73 and SG, to the more intense red and purple tones of RR and SN, respectively (Fig. 1a). A discrepancy was represented by the detection of anthocyanins in the colorless FC corncobs. Furthermore, in both RR and SN corncobs, despite their intense glume pigmentation, the levels of anthocyanins were comparatively lower than those reported in the literature for purple cobs of other maize varieties^30–32^. Unlike these varieties, where the corncob is entirely pigmented, in SN, as well as in the other landraces used in this work, pigments were exclusively present in the glumes, which constitute the outer layer (chaff) of the structure, representing approximately 20% of the corncob dry weight^33^.

In maize a specific branch of the flavonoids pathway exists, leading to the biosynthesis of phlobaphenes, complex molecules resulting from the polymerization of flavan-4-ols. Phlobaphenes, alongside anthocyanins, are accounted for the red/purple pigmentation observed in seed pericarps and corncob glumes. The biosynthesis of these compounds is regulated by the MYB transcription factor *pericarp color1* (*p1*) that leads to the production of the flavan-4-ol precursors, apiforol, and luteoforol^29,34^. Various *P1* alleles confer diverse pericarp and corncob glumes colors by controlling the preferential accumulation of luteoforol (resulting in a dark red hue) in comparison to apiforol (resulting in a light red hue), or viceversa^35^. A previous study on the SN maize genotype indicated a substantial accumulation of phlobaphenes in the seed pericarp, considered as the primary cause of the distinctive intense red pigmentation observed in the kernels. On the contrary, the anthocyanin content in this tissue was found to be minimal, similar to the levels detected in the SN corncob glumes in our study. The authors hypothesized that unpolymerized forms of phlobaphenes, rather than anthocyanins, might have been detected since flavan-4-ols can be present in methanolic pigment extracts^35, 36^. Furthermore, in purple corn a phenomenon known as co-pigmentation is reported, due to the non-covalent interaction between anthocyanins and other flavonoids, resulting in altered absorbance and hue effect^34^. Based on these findings, it cannot be excluded that also in the maize landraces used in the present work different phlobaphenes forms (either polymerized or not) may contribute to the pigmentation of corncob glumes. The observed variations in the color across the corncobs, as well as in the levels of detected pigments, might be attributed to an additive or interfering effect of phlobaphenes on anthocyanins. Further investigations are needed to validate these hypotheses.

In terms of the proximate composition of corncobs (Fig. 1b,c), the predominant component was the insoluble fraction of dietary fiber (IDF). This fraction accounted for an average of 86% of the corncob DW (Fig. 1c), while the level of the soluble fraction of fiber (SDF) was minimal. The contents of crude protein, sugars, starch, and ash in corncobs (Fig. 1b) exhibited significant variations among some of the five maize varieties: SN showed the highest level of crude protein and available carbohydrates (sugar plus starch), while B73 displayed the lowest level of available carbohydrates, and SG showed the lowest amounts of protein and ash. Collectively, these nutrients comprised 10–15% of corncob DW. Our findings are in line with those reported in the literature, which emphasize high fiber contents of approximately 90% (comprising cellulose, hemicellulose and lignin), along with comparable levels of ash and protein at 4% in the corncobs of different maize varieties, expressed on dry weight^37,38^. In contrast, we observed high levels of available carbohydrates (ranging from 4 to 11%), which could be attributed to the maturity stage of our samples^39^.

**Figure 2:**
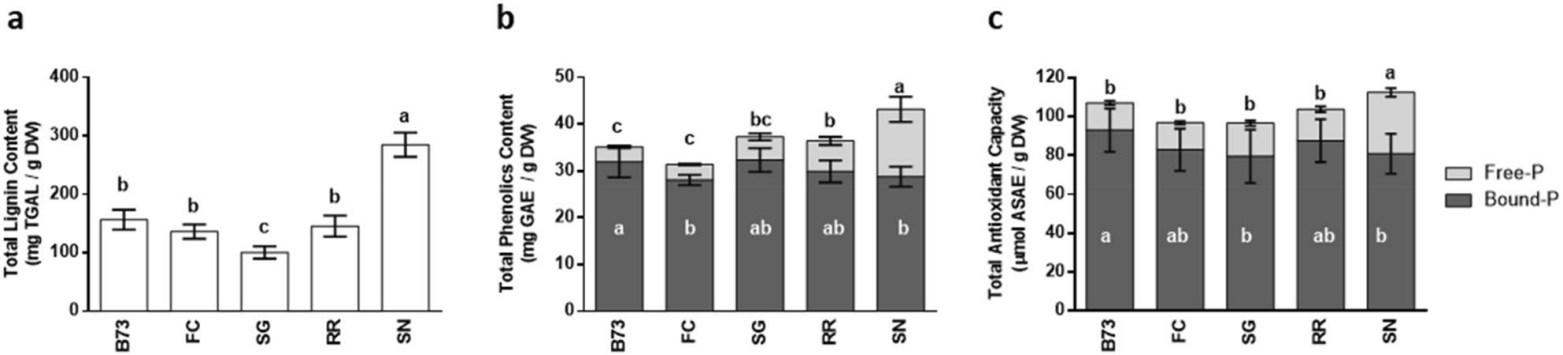
Contents of lignin and phenolics in corncobs. (**a**) Total lignin content (mg thioglycolic acid lignin/g DW), (**b**) total phenolics content (mg gallic acid equivalent/g DW) and (**c**) total antioxidant capacity (nmol ascorbic acid equivalent/g DW) in B73, ‘Fiorine di Clusone’ (FC), ‘Spinato di Gandino’ (SG), ‘Rostrato Rosso di Rovetta’ (RR), and ‘Spinoso Nero Valle Camonica’ (SN) corncobs. Bars represent the means ± SD (n = 3). Different letters indicate statistically significant differences (*P* < 0.05) among genotypes evaluated with two-way ANOVA.

The corncobs were further characterized for the total contents of lignin (Fig. 2a), free and bound phenolics (Fig. 2b) and total antioxidant capacity in the free and bound phenolic fractions (Fig. 2c). This comprehensive analysis provides, for the first time, a detailed description of corncobs of Italian maize landraces regarding these parameters.

The assessment of lignin content was conducted by the non-destructive method of thioglycolic acid derivatization^40^, instead of adopting the destructive gravimetric protocol commonly used for this material. The lignin contents, expressed as mg of lignothioglycolate derivatives (mg TGAL/g DW, Fig. 2a), varied among the different genotypes, and were low in SG (100.5 ± 11 mg TGAL/g DW), intermediate in FC, RR and B73 (136.3 ± 12, 145.4 ± 18, 157 ± 17 mg TGAL/g DW, respectively), and remarkably high in SN (285 ± 21 mg TGAL/g DW).

The three tissue fractions of the corncob, namely the chaff, woody ring, and pith, account for 21.1%, 77.5%, and 1.4% of the DW, respectively. The primary components within these fractions include hemicellulose (33–43%) and cellulose (26–36%), while lignin constitutes 17–21% of the DW, primarily distributed in the pith and chaff rather than the woody ring. Lignin is mainly associated with the middle lamella portion of the parenchyma cell wall in all corncob tissues, as well as with the vascular bundle cells of the woody ring^33^. Lignin, a complex aromatic heteropolymer, is synthesized through the phenylpropanoid metabolism. The cinnamic acid generated in the initial reaction of the pathway undergoes a series of reduction processes, ultimately leading to the production of hydroxycinnamyl alcohols (*p*-hydroxyphenyl, guaiacyl and syringyl), known as monolignols. These monolignols polymerize via the action of peroxidase and/or laccase enzymes, resulting in the formation of lignin^41^. Guaiacyl, syringyl, and *p*-hydroxyphenyl lignins are present to varying degrees in the different corncob fractions, associated with *p*-hydroxycinnamic acids such as ferulic (predominant in chaff and pith) and *p*-coumaric (predominant in the woody ring). This association facilitates the linkage of lignin to cell wall polysaccharides, thereby forming lignin-carbohydrate complexes. The lignin content and composition, along with the associated *p*-hydroxycinnamic acids, contribute to the recalcitrance of lignocellulosic biomass to hydrolysis, thus limiting the re-use and valorization of corncob waste residues^42^.

Concerning phenols, these compounds in plants are synthesized via the shikimate/phenylpropanoid pathway, leading to a multitude of intermediates with one or more aromatic rings coupled with single or multiple hydroxyl groups. Phenols are typically classified as phenolic acids, flavonoids, stilbenes, coumarins, and tannins. They occur in free soluble forms (aglycones) or as soluble conjugated (esterified to sugars or other low molecular mass compounds), as well as in insoluble bound forms (crosslinked to cell wall polymers). Flavonoids are generally free soluble, while high molecular mass phenolics, along with hydroxycinnamic acids (such as *p*-coumaric and ferulic acids) esterified to cell wall components, are insoluble^43,44^. In plants, they act as protectors against UV light, biotic stresses, and function as antioxidants, pollinator attractants, enzymes inhibitors, and allelopathic agents^43,45,46^. Our interest in these compounds relies on their beneficial effects on health, due to their antioxidant activity in scavenging free radicals, disrupting radical chain reactions, and chelating trace elements, thereby playing a role in the prevention of oxidation-linked chronic diseases^47,48^.

SN corncobs had the highest total phenolics content (43.2 ± 4.8 mg GAE/g DW), whereas FC and B73 corncobs showed lower values (33.2 ± 4 and 35.1 ± 3.6 mg GAE/g DW, respectively) (Fig. 2b). A substantial proportion (33%) of soluble free compounds was observed in SN, followed by SG and RR corncobs (16%), while the presence of free compounds in B73 and FC corncobs accounted for only 9% of the total phenolics. Regarding the bound phenolics content, the highest values were detected in SG and B73 corncobs (32.3 ± 2.5 and 32 ± 3.3 mg GAE/g DW, respectively), followed by FC and RR (29.9 ± 3 mg GAE/g DW), and SN (28.8 ± 2.1 mg GAE/g DW) corncobs.

The highest antioxidant capacity was observed in the insoluble bound phenolic extracts of B73 and RR corncobs (93 ± 11 and 88 ± 11 µmol ASAE/g DW, respectively), which exhibited the most significant values, followed by FC, SN and SG corncobs (83 ± 11, 81 ± 10 and 80 ± 14 µmol ASAE/g DW respectively) (Fig. 2c). In contrast, the antioxidant capacity in the free phenolic extracts was relatively lower, although resulted twice as high (32 ± 2.2 µmol ASAE/g DW) in SN corncobs as in those of all the other genotypes (14.0 ± 0.8, 14.1 ± 1.0, 16.2 ± 1.5 and 17 ± 1.4 µmol ASAE/g DW for FC, B73, RR and SG, respectively).

The levels of total phenolics (free + bound) detected in this study were comparable to the values reported in corncobs of the purple maize variety ‘Canteño’, which ranged between 38 and 50 mg GAE/g DW, depending on the specific growth location^49^. In this variety, the level of free phenolics, accounting for 77–86% of the total content, was strongly associated with the total monomeric anthocyanin contents (2000–3000 mg C3GE/100 g DW), which also showed the highest antioxidant capacity among the phenols. Similarly, in our experimental material, despite the greater abundance of bound phenols responsible for the majority of the antioxidant capacity, the free phenolics contents appeared consistent with those of anthocyanins in RR and, to higher extent, in SN corncobs. The flavonoids present in these corncobs may be accountable for the significantly high free radical antioxidant activity.

Overall, our data demonstrate the presence of variability, albeit to varying extent, among the different genotypes for all the examined traits.

### Solid state fermentations (SSFs)

In a preliminary screening, where the growth of MMs was evaluated through visual scoring (analysis) after 20 days of incubation, corncobs from the inbred line B73 supported the most robust fungal growth, regardless the MM strain used, whereas SN corncobs resulted the least performing substrate for MMs growth (Table 1). Regarding the MMs, *Pleurotus* (P) exhibited the most extensive growth, displaying a highly compact growth (+ + +) in B73 and a compact growth (+ +) in all substrates, followed by *Ganoderma* (G), which showed a compact growth (+ +) in all substrates except for SN, where the growth was superficial ( +). Consequently, these two MMs were selected for the subsequent phase of the study, wherein the MM growth on the SSF surface was monitored at various time points by means of Image Analysis, and the data were recorded as the percentage of Mycelial Growth Area (MGA).

**Table 1:** Growth of the tested Medicinal Mushrooms as determined through visual analysis after 20 days of incubation onto B73, ‘Fiorine di Clusone’ (FC), ‘Spinato di Gandino’ (SG), ‘Rostrato Rosso di Rovetta’ (RR), and ‘Spinoso Nero Valle Camonica’ (SN) corncobs.

Medicinal Mushrooms (MMs)	Corncob genotype				
	B73	FC	SG	RR	SN
*Pleurotus*	+++	++	++	++	++
*Ganoderma*	++	++	++	++	+
*Flammulina*	++	++	++	+	+
*Lentinula*	++	+/−	+/−	+/−	+/−

(+++) very compact growth, mycelium thickness around 2–3 mm, penetrating also on corncobs; (++) compact growth, mycelium thickness around 1 mm; (+) superficial growth, mycelium thickness lower than 1 mm; (+/−) thin superficial growth only.

**Figure 3:**
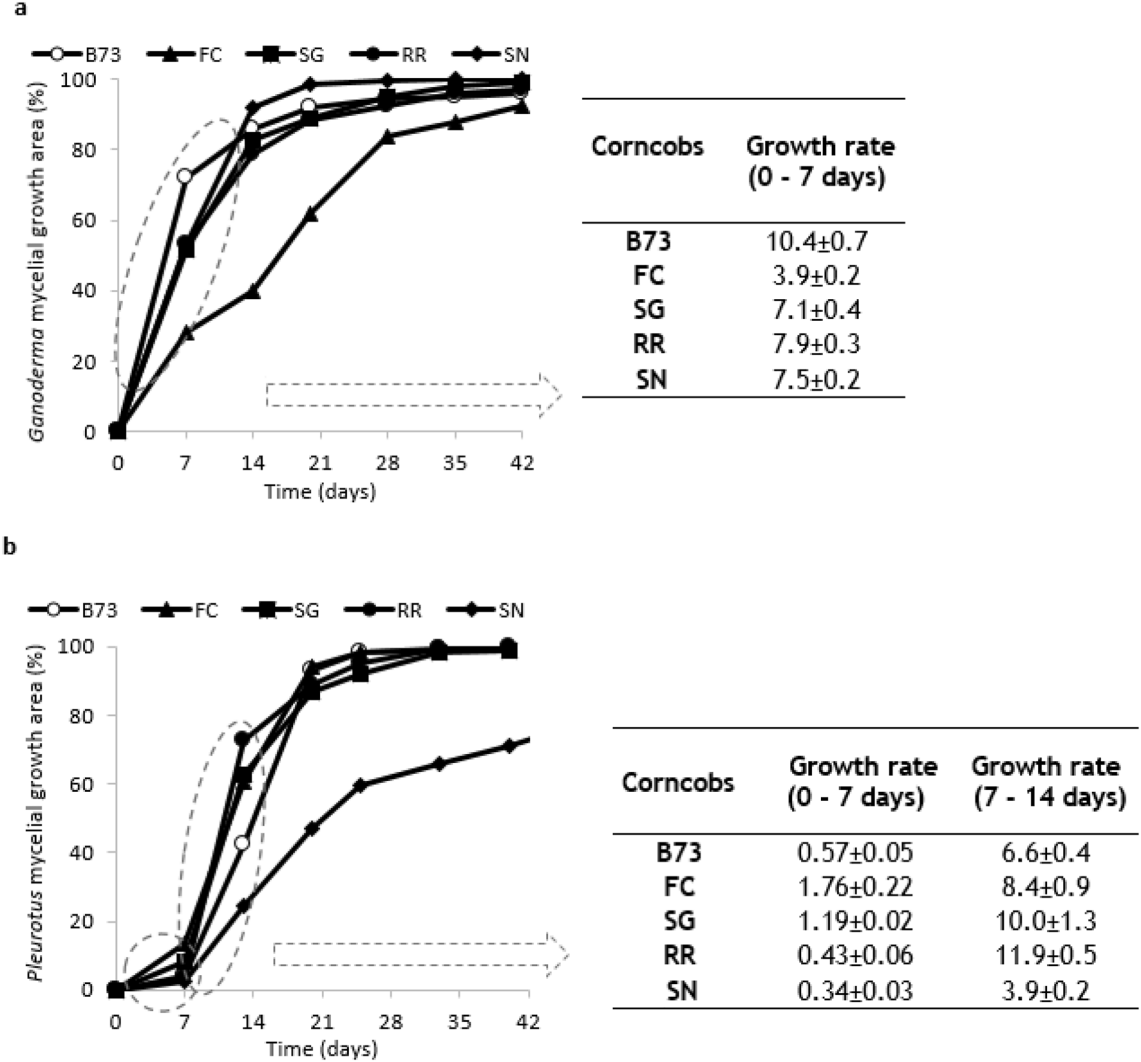
(**a**) *Ganoderma* and (**b**) *Pleurotus* mycelial growth area (MGA, %) over time (days) and data extrapolated from the growth curves obtained by SSFs Image Analysis, as the slope (angular coefficient) of the linear curve in the interval 0–7 days for *Ganoderma*, as well as 0–7 and 7–14 days for *Pleurotus*. MGA values reported as means (n = 2), CV ≤ 10; slopes reported as means ± SD (n = 2).

In the G growth profiles, no lag phase was observed, as the mycelial growth occurred immediately (Fig. 3a). This behavior may be attributed to the presence of constitutive enzymes in G capable of degrading lignocellulosic materials, as previously reported in a separate study^50^. The G strain always achieved approximately 92–100% surface coverage (Fig. 3a), indicating its ability to grow on all the tested corncobs. The type of corncobs noticeably affected the Mycelial Growth Rate, with the highest value (10.4%MGA/week) recorded on B73 and the lowest value (3.9%MGA/week) on FC corncobs.

In the P growth profile, a lag phase was instead observed, as the MGR was lower than 1.8% MGA/week during the first week (Fig. 3b). This behavior could be attributed to the presence of inducible enzymes responsible for the degradation of the lignocellulosic material^51^. In the second week, also the P MGR was influenced by the type of corncob: the fastest growth (11.9%MGA/week) was evidenced on RR corncobs, whereas the slowest growth (3.9%MGA/week) occurred on SN corncobs. In the latter case, the mycelial growth covered only 80% of the available surface, even when the incubation time was extended up to 75 days (data not shown).

### Characterization of culture samples obtained by SSF

After incubation, moisture content of culture samples obtained applying SSF process was approximately 0.6 g/g. Subsequently, the sample were dried at low temperature to achieve a final moisture of approximately 0.15 g/g and ground for the subsequent analyses.

Regarding the proximate composition, mycelial growth significantly influenced nutrient levels in cultures obtained by SSFs (Table 2). The percentage of protein increased by 21–71% following fermentation with P and from 31% to more than two-fold increment with G, compared to the relative corncobs alone. Similar increases in protein content following fungal growth on vegetable waste have been previously observed^52,53^ as well as with corncobs^54,55^. This rise in protein content could be attributed to the mushroom, which is considered a rich source of protein^56^, as well as to the modification of fiber-carbohydrate fractions, which might partially explain the increase of crude protein percentage, when expressed on dry basis. Furthermore, it is noteworthy that this result corresponds to an increase in the total nitrogen content in cultures obtained via SSFs, at least in part due to the nitrogen content of the mycelial cell wall, which includes chitin contents (*N*-acetylglucosamine)^57^.

**Table 2:** Chemical composition of corncobs and of corncob-based SSFs in the presence of *Pleurotus* (+ P) or *Ganoderma* (+ G). Values are expressed as g/100 g DW.

Corncob genotype	MM	Crude Protein	Sugar	Starch	Ash	IDF	SDF	β-glucan
B73	-	3.2 ± 0.1^c^	2.4 ± 0.1^a^	1.5 ± 0.3^a^	2.0 ± 0.0^b^	90.9 ± 0.2^a^	nd	nd
	+ P	3.9 ± 0.1^b^	0.2 ± 0.0^c^	0.7 ± 0.0^ab^	3.4 ± 0.6^a^	88.7 ± 0.3^b^	1.4 ± 0.0^b^	0.14 ± 0.02^a^
	+ G	4.2 ± 0.1^a^	0.4 ± 0.0^b^	0.1 ± 0.0^b^	3.4 ± 0.3^a^	83.3 ± 0.5^c^	4.3 ± 0.0^a^	0.24 ± 0.05^a^
FC	-	3.0 ± 0.0^c^	3.1 ± 0.0^a^	2.5 ± 0.6^a^	2.9 ± 0.1^b^	88.3 ± 0.6^a^	0.2 ± 0.0^c^	nd
	+ P	4.0 ± 0.0^b^	0.3 ± 0.0^b^	0.7 ± 0.0^b^	3.4 ± 0.1^b^	87.2 ± 0.3^a^	1.9 ± 0.0^b^	0.19 ± 0.01^a^
	+ G	4.4 ± 0.1^a^	0.2 ± 0.0^b^	nd	4.3 ± 0.1^a^	84.1 ± 0.5^b^	3.8 ± 0.0^a^	0.15 ± 0.07^a^
SG	-	2.4 ± 0.1^c^	6.6 ± 0.1^a^	4.0 ± 0.9^a^	1.7 ± 0.0^a^	85.1 ± 0.7^b^	0.1 ± 0.1^a^	nd
	+ P	4.2 ± 0.2^b^	0.3 ± 0.0^c^	0.8 ± 0.0^b^	2.3 ± 0.1^a^	88.3 ± 0.1^a^	1.1 ± 0.0^b^	0.16 ± 0.01^a^
	+ G	4.6 ± 0.1^a^	0.5 ± 0.0^b^	0.1 ± 0.0^b^	2.4 ± 0.2^a^	82.9 ± 0.5^c^	3.3 ± 0.0^a^	0.22 ± 0.04^a^
RR	-	2.6 ± 0.1^c^	6.7 ± 0.1^a^	2.7 ± 0.6^a^	2.4 ± 0.1^b^	85.3 ± 0.8^a^	0.2 ± 0.2^c^	nd
	+ P	4.4 ± 0.1^b^	0.2 ± 0.1^a^	0.6 ± 0.0^b^	3.5 ± 0.5^a^	85.6 ± 0.8^a^	1.8 ± 0.0^b^	0.21 ± 0.03^a^
	+ G	5.3 ± 0.1^a^	0.4 ± 0.0^b^	0.1 ± 0.0^b^	3.5 ± 0.1^a^	81.5 ± 0.3^b^	5.0 ± 0.0^a^	0.27 ± 0.09^a^
SN	-	4.2 ± 0.0^c^	5.7 ± 0.1^a^	5.5 ± 0.2^a^	2.2 ± 0.1^b^	81.7 ± 0.2^b^	0.7 ± 0.1^c^	nd
	+ P	5.3 ± 0.0^b^	1.4 ± 0.0^b^	0.8 ± 0.0^b^	2.7 ± 0.0^ab^	82.6 ± 0.0^ab^	1.6 ± 0.0^b^	0.17 ± 0.02^b^
	+ G	5.9 ± 0.1^a^	0.2 ± 0.0^c^	nd	3.3 ± 0.1^a^	84.3 ± 1.5^a^	3.6 ± 0.0^a^	0.37 ± 0.07^a^

Data (means ± SD) not sharing common letters within the same variable and corncob genotypes are significantly different (*P* < 0.05). See Figs. 1a and 5 for sampling codes of the corncob genotypes and MMs strains.

*IDF* insoluble dietary fiber; *SDF* soluble dietary fiber; *nd* not detectable; (-) corncob alone.

In contrast, available carbohydrates were significantly reduced following MMs fermentation, as they were metabolized as carbon and energy sources: free sugars were reduced by approximately tenfold, and starch levels decreased to undetectable levels, as observed in FC + G and SN + G, compared to corncobs. Additionally, G growth led to a decrease in IDF in all corncobs, except SN (Table. 2), while P resulted less effective in reducing IDF. Simultaneously, detectable amounts of SDF, which were initially negligible in corncobs, were found after MMs growth: the SDF contents following P growth ranged between 1.1 and 1.9 g/100 g DW, and G further increased SDF levels to 3.3–5.0 g/100 g DW. Fermentation with MMs also resulted in higher levels of β-glucan compared to corncobs alone, with a significant distinction observed in the SN sample. Following fermentation by mushroom growth, a similar increase in β-glucan was also found in other agricultural waste and mycelia mixture^58^. However, the enrichment of β-glucan in our culture samples was relatively low. Although the fungal cell wall contains this fiber, the quantity assessed in the mycelium is generally lower than that in the fruiting body^59^. Furthermore, in terms of dry weight, the contribution of the mycelium to the SSF culture sample, compared to the corncob substrate, is lower; both those conditions could contribute to the results.

Overall, these findings suggest that MMs growth altered the cellulosic structure of corncobs, affecting the contents of dietary fiber fractions, and that G growth proved to be more effective.

**Figure 4:**
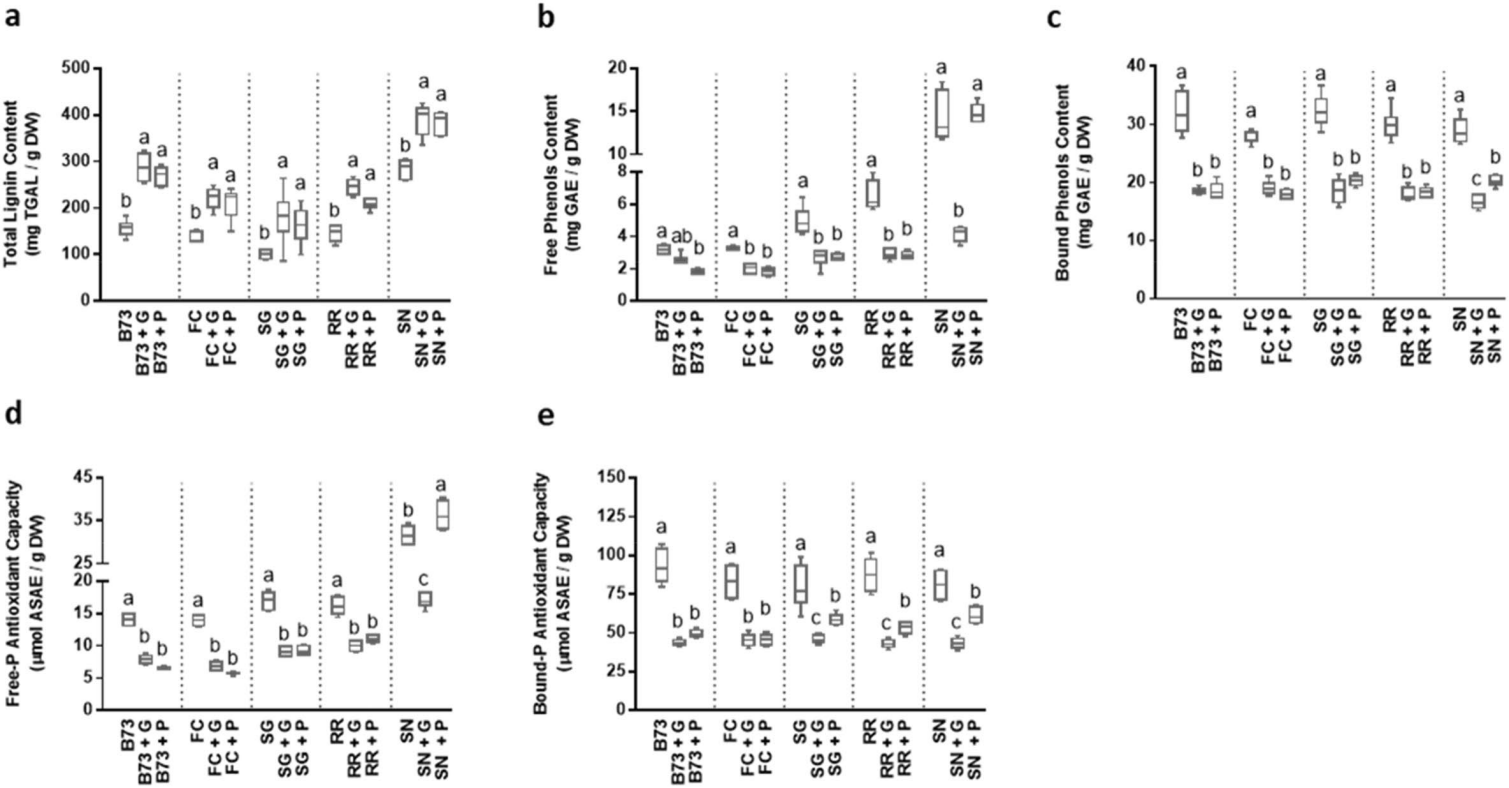
Effect of the growth of *Ganoderma* (+ G) or *Pleurotus* (+ P) on corncob traits. (**a**) Total lignin content (mg thioglycolic acid lignin/g DW), (**b**) Free Phenols and (**c**) Bound Phenols content (mg gallic acid equivalent/g DW), (**d**) Free-P and (**e**) Bound-P antioxidant capacity (nmol ascorbic acid equivalent/g DW) in SSFs based on B73, ‘Fiorine di Clusone’ (FC), ‘Spinato di Gandino’ (SG), ‘Rostrato Rosso di Rovetta’ (RR), and ‘Spinoso Nero Valle Camonica’ (SN) corncobs. Values represent the means ± SD (n = 3). Two-way ANOVA (*P* < 0.05) was performed to evaluate statistically significant differences within the same maize genotype.

In all culture samples obtained using SSF processes, the total lignin contents were significantly higher compared to those measured in the corncobs. In FC-, B73- and SG-based culture samples, with both G or P, the lignin levels (Fig. 4a) were 63–78% higher than those in the respective corncobs, reaching values of 222 ± 20, 278 ± 24 and 179 ± 19 mg TGAL/g DW, respectively. In RR-based samples, the total lignin contents detected were 245 ± 17 mg TGAL/g DW after the growth of G and 208 ± 11 mg TGAL/g DW after the growth of P, representing an increase of 69% and 43% respectively, compared to the corncobs alone. When both MMs were grown on SN-based cultures the total lignin contents detected were (only) 36% higher than those measured in the respective corncobs.

The higher lignin contents detected in cultures samples compared to corncobs might be attributed to the fungal degrading action of the corncob substrate, leading to a relaxation of the cell wall structure so causing a greater exposure of the pectic compounds along with the associated lignin fraction trapped inside. This hypothesis is supported by the data obtained in the proximate analyses (Table. 2), which show that the MMs growth was accompanied by a slight but significant decrease in IDF, mainly comprising the holocellulose components of the fungal cultures, and an increase in SDF, representing the pectic fraction. This effect, though more pronounced for *Ganoderma* than *Pleurotus*, indicates that the mushrooms promoted the breakdown of the cell wall structure. Their degrading activity is also suggested by the significant decrease in the bound phenolic content (Fig. 4c) observed in the different corncob-based substrates during the MMs growth.

The consumption of insoluble phenol compounds, such as the phenolic acids capable of linking lignin to the hemicellulose matrix, may have led to the detection of higher amounts of thioglycolic acid lignin. The two MMs used belong to the group of the white rot basidiomycetes, which are considered the most efficient organisms at degrading lignocellulose components due to their ability to secrete lignin oxidizing and holocellulose-hydrolyzing enzymes, a set of proteins known as secretome^60,61^. Specifically, lignin consumption is accomplished by phenol oxidases (laccases) and heme-peroxidases [manganese-dependent peroxidase (MnP), lignin peroxidase (LiP), versatile peroxidase (VP)], as well as by accessory enzymes such as H_2_O_2_-generating oxidases and non-enzymatic mechanisms that result in the production of free hydroxyl radicals^61^. General nutrient-sensing pathways that prioritize the use of preferred carbon sources are reported to control the activation of genes encoding the secretome and to inhibit the energy-intensive production of plant biomass-degrading enzymes^62^. Under our conditions, both MMs showed nearly complete consumption of sugars and starch (Table. 2). The availability of these easily accessible carbon and energy sources might have affected the secretome expression, so limiting ligninolysis. The composition and activity of secretome, whose expression can vary in a temporal sequence according to the phase of degradation of lignocellulose^63^, seem to depend on fungal strain as well as substrate nature. A recent study indicated that for *P. ostreatus,* laccase activity appears to be substrate-dependent^64^.

**Figure 5:**
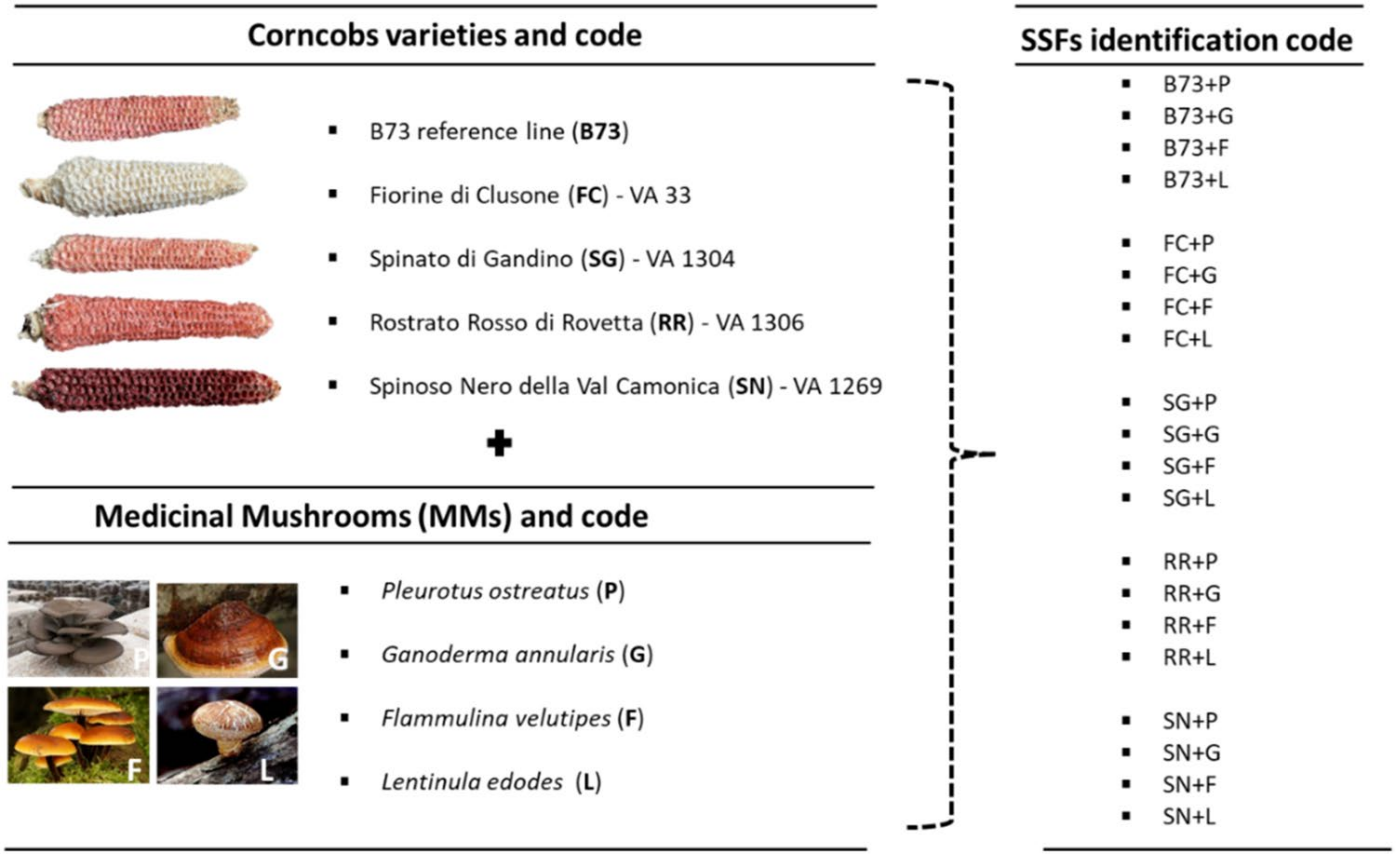
Identification and sampling codes of the corncob genotypes, the Medicinal Mushrooms (MMs) strains and of the Solid State Fermentations (SSFs) set-up in the present research project.

The growth of MMs significantly reduced the total phenolics contents by an average of 40% compared to the values measured on corncobs alone (Fig. 4b,c). The only exception was represented by the free phenols in SN + P, where levels (14.4 ± 0.8 mg GAE/g DW) did not change compared to that of the control corncob. However, even in this condition, the total (free + bound) phenolics content was lower (− 20%) in culture samples than in SN control corncob. Conversely, in the SN + G sample, a significant strong decrease (− 71%, respect to the control) in free phenols content was measured. A similar trend of changes (− 44%, as average, compared to corncobs alone) was observed for the total antioxidant capacity evaluated in both phenol fractions (free and bound: Fig. 4d,e) from the different corncob-based culture samples in the presence of both MMs. In particular, the value of this parameter in the free phenols fraction from SN + P was 36.3 ± 3.4 µmol ASAE/g DW, resulting even higher (+ 15%) than that of SN- control corncob. The presence of *Ganoderma* significantly reduced the antioxidant capacity in the free phenol fraction of SN (Fig. 5d) and in the bound phenol fractions of SG-, RR- and SN-based culture samples (Fig. 5e).

Studies conducted on basidiomycetes and ascomycetes strains grown using SSF processes on various substrates indicated that the growth of basidiomycetes was associated with the consumption of phenolic compounds^65^. These strains showed a low capacity to produce phenolics but a great ability to consume those present in the substrate, mainly through the action of peroxidase and phenol oxidases. A variable behavior was observed in this taxonomic group, with *Ganoderma* spp. showing a great ability to both intensely produce and consume phenolics^64, 65^. This might partly explain the different growth rates of *Ganoderma* and *Pleurotus* observed within the first period (14 days) of cultivation on the different corncobs considered in the present work (Fig. 3a,b).

In our conditions, the growth of the two MMs on the different corncob-based substrates was accompanied by a strong decrease in the total free and bound phenols contents and in their relative DPPH scavenging activity. The only exception was the sample in which *Pleurotus* was inoculated onto SN, where the reduced MM growth (Fig. 3b) was accompanied by the lack of phenols and DPPH scavenging activity consumption (Fig. 4b,e). Consistent with results in the literature^65^ indicating that moderate concentrations of phenols in the substrate might sustain the fungi POX activity, whereas high phenolic concentrations inhibit it, the observed effect of the substrate on the *Pleurotus* growth might possibly be ascribable to the very high content of free phenols that characterizes the intensely pigmented SN corncob compared to the others. Some species of *Pleurotus*, which have been reported to have a high ability to consume phenolics, proved unable to consume both these substances and the partially correlated DPPH scavenging activity when grown on the flavonoid-rich pomegranate peel substrate^65^.

The different availability of phenolics in substrates could modulate the growth response of *Pleurotus* and *Ganoderma*, as suggested by the slow growth rate of *Ganoderma* on FC corncob. This substrate, besides having low sugar and starch levels, is characterized by the lowest content of total phenolics. The combined reduced availability of these compounds may have possibly affected the prompt development of the mycelium.

Overall, the obtained findings suggest the possibility of valorizing these cultures obtained applying SSF processes within the circular economy; ongoing studies are exploring the utilization of this biomass as a new ingredient for the production of gluten-free baked goods.

## Conclusions

In this study we characterized the growth pattern of MMs on substrates containing corncobs, derived as residues from the cultivation of different maize genotypes. We compared the growth of *Pleurotus ostreatus* and *Ganoderma annularis* on corncobs with distinct composition and demonstrated that MMs growth induced changes to the corncob composition.

Based on the analysis of the Mycelial Growth Rate (Table1 and Fig. 4) we observed that both *Ganoderma* and *Pleurotus* were capable to grow on all the tested substrates and in most substrates reached 92–100% surface coverage within 42 days. However, *Pleurotus* showed the lower growth rate and reached a lower surface coverage on SN corncobs (Fig. 4b), on which it never reached the full substrate colonization within the duration of the assay. We may speculate that the high content of SN free phenols may have exerted an inhibitory effect on *Pleurotus* growth. This is supported by the observation that, differently from what observed in the other cob-fungus combinations, the free phenol content did not decrease following *Pleurotus* growth on this substrate (Fig. 4b). Overall, these observations suggest that, in addition to carbohydrates, phenols could play a crucial role in MMs growth, and the differential availability of these secondary compounds might be differently perceived by the fungal strain employed. Further investigations are required to confirm these hypotheses and elucidate the nature of the soluble phenolic fraction, along with other soluble compounds, and their influence on *Pleurotus* growth.

Interestingly, *Ganoderma* achieved a highest surface coverage in the shorter duration when grown on SN, whose distinct features are the highest protein, starch (Fig. 1b) and lignin (Fig. 2a) contents. The *Ganoderma*-SN corncob combination emerges as the most favorable choice in terms of fungus growth. However, considering the high lignin content in the SN based-culture and the possibility of obtaining interesting amounts of β-glucans, the *Ganoderma*-RR combination will be preferred for future applications on food product.

Further studies should be performed to investigate the corncob-mushroom interactions. For instance, variants in regulatory genes involved in the general phenylpropanoid pathway, from which lignin is produced^66–68^, could be utilized to set up different corncob-based SSFs and test the effect of these changes on *Ganoderma* growth and the nutritional properties of culture samples obtained by SSF. Based on these findings, the phenylpropanoid pathway could be manipulated in cultivated maize genotypes to improve the exploitation of lignin in corncob waste. An in-depth investigation on composition and activity of MMs secretome could also help to individuate the most efficient strains to be used for corncob waste valorization through SSF process.

Overall, the proposed biotechnological approach was effective in converting a by-product (corncob) into a matrix rich in soluble fiber, thereby opening up new opportunities for the utilization of corncobs as an active ingredient to enhance the nutritional value of food preparations. The use of MMs has contributed to the biotechnological valorization of corncobs, enabling the production of high-end products through green and ecofriendly processes, fostering environmental sustainability and promoting circular bioeconomy practices.

## Methods

### Chemicals

All chemicals as well as lignin, the Folin-Ciocalteu phenol reagent, 2,2-diphenyl-1-picrylhydrazyl (DPPH), gallic acid (GA), ascorbic acid (ASA), thioglycolic acid (TGA) and Triton X-100 were purchased by Sigma Aldrich. The culture medium Potato Dextrose Agar (PDA) was purchased by Formedium (Swaffham, England). K_2_HPO_4_, (NH_4_)_2_SO_4_ and MgSO_4_ were from VWR International srl (Milano, Italy). Yeast extract was purchased by Costantino SpA (Torino, Italy) while glucose by Duchefa (Haarlem, the Netherlands).

### Plant materials

Five maize (*Zea mays* L. ssp. mays) genotypes (Figs. 1, 5) were adopted as sources of corncobs. These genotypes include the reference inbred line B73^69^, and four distinct maize landraces, namely Fiorine di Clusone (FC), Spinato di Gandino (SG), Rostrato Rosso di Rovetta (RR), and Spinoso Nero Valle Camonica (SN). The seeds of the B73 reference inbred line were originally provided by the Maize Genetics Cooperation Stock Center (http://maizecoop.cropsci.uiuc.edu), while the seeds of the four maize landraces FC (VA 33), SG (VA 1304), RR (VA 1306) and SN (VA 1269) were originally provided by the germplasm bank of CREA Bergamo (www.ecpgr.cgiar.org/working-groups/maize/maize-wg). Plants were subsequently propagated via sibling mating in the open experimental field at the University of Milan. At the conclusion of the growing season (2021), ears were manually harvested, shelled, and dried to achieve a moisture content of 12–13%. Following seeds removal, the corncobs were stored at room temperature until research analyses.

The authors confirm that the use of plants in the present study complies with international, national, and/or institutional guidelines.

### Microbial strains

The study utilized the following fungal strains (Fig. 5): *Pleurotus ostreatus* ATCC 96997 (ATCC: American Type Culture Collection, Manassas, VA, USA), *Ganoderma annularis* DSMZ 9943 (DSMZ: Deutsche Sammlung von Mikroorganismen und Zellkulturen GmbH, Braunschweig, Germany), *Flammulina velutipes* MIM 82 (MIM: Microbiologia Industriale Milano, Italy), and *Lentinula edodes* CBS 454.69 (CBS: Centraalbureau voor Schimmelcultures, Utrecht, The Netherlands). All the strains were maintained on 5 cm plates containing PDA culture medium. Inoculum was performed by depositing a quarter (around 5 cm^2^) of an older (max 2 months) solid culture plate to the surface, using a sterile scalpel. The plates were subsequently incubated at 25 °C in the dark. When the mycelium completely covered the surface of the solid culture, the plates were tightly sealed with Parafilm and stored at 4 °C for a maximum of 2 months before use.

### Solid state fermentation (SSF) trials: screening

Preliminary trials applying the SSF technique were carried out using Pyrex glass containers with screw caps, each with a total volume of 250 mL. The corncobs were initially roughly crushed with a knife and then further ground to a maximum size of 2 mm using a Thermomix 300 (Worwerk, Wupperthal, Germany) at speed 7 for 1 min. Seven grams of the ground corncobs were then placed in each glass container and sterilized at 117 °C for 20 min. After cooling to room temperature, the corncobs were supplemented with 14 mL of a previously sterilized mineral solution containing (g/L): K_2_HPO_4_ 1, (NH_4_)_2_SO_4_ 5, MgSO_4_ 0.2, yeast extract 1, and adjusted to a pH of 5.8. Each culture was inoculated with a pre-grown MM solid culture prepared as previously described on a 5-cm PDA plate, taken off and chopped with a sterile scalpel. The cultures were all incubated at 25 °C in the dark, with the mycelium development being monitored throughout the incubation period.

### Evaluation of mycelial growth

Based on the results of the first screening carried out in SSF, *Ganoderma* (G) and *Pleurotus* (P) MMs were selected for subsequent research steps. These two fungal strains were cultivated using SSF employing the five studied corncobs varieties. Containers suitable for fungal growth (16 × 10 cm) equipped with a filtering air system (Berry Superfos, Taastrup, Denmark) were filled with 50 g of ground corncobs, which were sterilized and then soaked with 100 mL of the mineral solution, as already described during the screening. The cultures were inoculated with 20 mL of a liquid fungal pre-culture, prepared as follows: a quarter (around 5 cm^2^) of a pre-grown solid culture (not older than 2 months) on a PDA plate was excised with a sterile scalpel and inoculated into 500 mL Erlenmeyer flasks containing 100 mL of a sterile liquid medium with the same composition as the mineral solution, supplemented with 20 g/L glucose. The flasks were incubated at 25 °C on an alternative shaker (40 spm, 4 cm run) in the dark for 7 days to obtain a visible growth (presence of pellets). These glucose-grown liquid pre-cultures were then employed as inoculum for the SSFs cultures.

**Figure 6:**
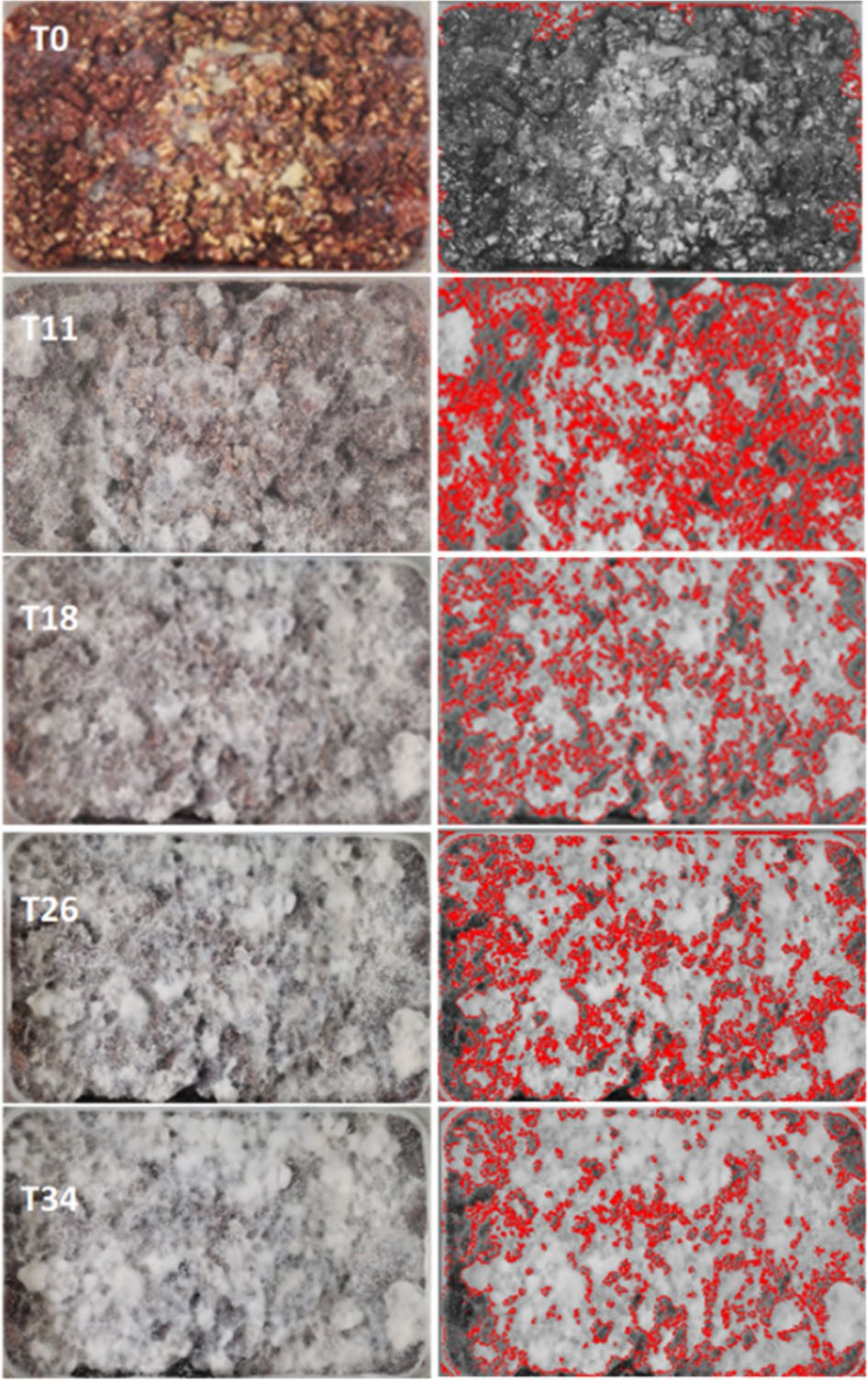
Example of mycelial growth (white area; left) over time (from 0 to 30 days, approximately) and image editing and elaboration (right).

The growth of MMs was monitored for at least 42 days by acquiring the image of the surface of the SSFs cultures (Fig. 6) once a week. A PowerShot G7X Mark II (Canon, Europe) camera was employed to capture the images under fixed conditions, including light exposition and a constant camera-to-sample distance. Image-Pro Plus 6.0 (Mediacybernetics, Maryland, USA) software was used to edit and process the images, i.e., converting to greyscale and adjusting the intensity range to identify and quantify the total box area (mm^2^) and the Mycelial Growth Area (MGA, mm^2^). The percentage of growth (MGA, %) was calculated as (MGA/box area) × 100, while the growth rate (MGR, %MGA/week) was derived from the growth curves as the slope (angular coefficient) of the initial linear curve within the time frame of 0 to 7 days for *Ganoderma* and 0 to 7 and 7 to 14 days for *Pleurotus*.

### Samples (corncobs and cultures) stabilization

To stabilize the culture samples and prevent mycelia overgrowth without excessively affect polyphenols content, after incubation the samples were dried at low-temperature (50 ± 2 °C) in a vacuum oven (WIPA, GEASS, Turin, Italy; operating at 0.987 Pa) to reach a moisture content of approximately 0.15 g/g. Since the resulting culture samples were intended for use as new valuable ingredients in food application, their particle size was subsequently reduced as much as possible, considering a balance between the finest particles achievable with laboratory mills and the typical particle size of traditional and unconventional flours^70^. To achieve this, both the dried corncobs and the culture samples were ground at room temperature using a disc-mill (MLI 204, Buhler, Italy) to pass through a screen with 1.0-mm openings. A minimum of four milling steps were necessary (setting the disc-mill at 8, 4, 2 and 0 levels) to produce powders with a particle size ≤ 1.0 mm without any sample loss (sieve retentate < 1%). The resulting powders were packed in plastic bags and stored in the dark at 4 °C until their characterization.

### Chemical composition

The proximate analyses were carried out in triplicate on powdered samples, following established official methods^71^. The determination of total starch content was performed using the AACC Method 76–13.01, by using the K-TSTA kit (Megazyme, Wicklow, Ireland). Free sugars were assessed by HPLC, according to Rocklin and Pohl^72^. The analysis of soluble and insoluble dietary fiber was carried out by the Prosky method (AACC n° 32–07.01): the content of β-glucan in the soluble dietary fiber fraction was evaluated by the K-YBGL 07/20 kit (Megazyme, Wicklow, Ireland).

### Extraction of anthocyanin compounds from corncobs and total monomeric anthocyanin contents determination

Anthocyanin compounds were extracted from powdered samples according to the methods described in literature^31,49^. The extraction process was conducted in triplicate. Briefly, 0.1 g of the sample was extracted with 5 mL of 0.01% (v/v) 6 N HCl acidified 50% aqueous ethanol (solid–liquid ratio 1:50, w/v). The extraction was performed under agitation in a rotary shaker (Tovamed, Montreuil, France; 200 rpm) at room temperature (RT) in the absence of light, overnight. The slurry was then centrifuged at 5000 ×*g* for 20 min (centrifuge Eppendorf 5804 R, Milan, Italy), and the resulting supernatant was stored at − 20 °C until analysis.

The determination of total monomeric anthocyanin contents was accomplished using the pH differential method^73^. The extracts were diluted with 0.025 M KCl (pH 1.0) or 0.4 M Na-acetate (pH 4.5), and the absorbance of each diluted extract was measured at 520 and at 700 nm using an UV–Visible spectrophotometer (UviLine 9400 Secomam, Thermo Fisher Scientific, Monza, Italy). Total monomeric anthocyanin contents, expressed as cyanidin-3-glucoside equivalents (C3GE) per 100 g dry weight, were calculated using the following equation:

$${\text{Anthocyanin pigment }}\left( {{\text{mg}}/{1}00 {\text{g}}} \right)\, = \,{\text{AB}}/\varepsilon {\text{L}} \times {\text{MW}} \times {\text{DF}} \times {\text{V}}/{\text{G}} \times {1}00$$

where AB is the absorbance calculated as (A520–A700 nm) pH 1.0—(A520–A700 nm) pH 4.5; ε is cyanidin-3-glucoside molar extinction coefficient [26900 L/(mol × cm)]; L is the cell path length (1 cm); MW is the molecular weight of cyanidin-3-glucoside (449.2 g/mol); DF is the dilution factor; V is the final volume (mL); G is the dry weight (g)^74^.

### Extraction of the free and bound phenolic fractions from corncobs and culture samples and total phenolic contents determination

The extraction of free and bound phenolics from the powdered samples was optimized using the methods reported by Dinelli et al.^45^ and Ranilla et al.^49^. The extraction was performed in triplicate. Aliquots of 0.2 g sample were extracted with 4 mL (solid–liquid ratio, 1:20) of 0.1% HCl methanol/acetone/water (45:45:10, v/v/v) under agitation in a rotary shaker for 1 h at RT in the dark. The resulting slurry was then centrifuged at 8,000 × g (Sorvall RC-5B, DuPont Instruments, Milano, Italy) for 30 min at 4 °C, and the obtained pellets were subjected to re-extraction under the same conditions. The recovered supernatants containing the free phenolic fraction were combined, vacuum-evaporated to dryness at 45 °C and reconstituted with a minimum volume (approximately 2 mL) of 70% (v/v) methanol. The final residue obtained after the second extraction of free phenols was collected and resuspended in 6 mL of 2 N NaOH. Alkaline hydrolysis was performed with agitation in a rotary shaker for 2 h at RT in the dark. The solution was then adjusted at pH 2 by the addition of concentrated HCl. Bound phenolics were then extracted three times with 6 mL of a mixture of cold mixture of diethyl ether (DE) and ethyl acetate (EA, 1:1, v/v) through manual shaking and subsequent centrifugation (5,000 × g for 20 min at 4 °C). The DE/EA fractions were combined, vacuum-evaporated to dryness at 45 °C, and dissolved in a minimum volume (ca. 1 mL) of 70% (v/v) methanol.

The total phenolic contents were determined in both free and bound phenolic fractions extracted from the dried and milled samples using the Folin-Ciocalteu method with some modifications^75^. In this method, 20 mL of the extract was mixed with 0.1 mL of Folin-Ciocalteu phenol reagent (1:1) and 1.58 mL of distilled water. The samples were vortexed, and after 5 min, 0.3 mL of a 20% (w/v) Na_2_CO_3_ solution were added. The mixture was then thoroughly shaken and then allowed to incubate for 1 h at RT in the dark. The absorbance was subsequently measured at 765 nm. The results were expressed as mg of gallic acid equivalents (GAE)/g DW. A calibration curve was plotted by mixing increasing aliquots (ranging between 3 and 50 mg in 20 mL) of GA in 70% methanol with the reaction solution.

### Antioxidant capacity determination in free and bound phenolic fractions from corncobs and culture samples

The antioxidant capacity of the free and bound phenolic fractions powdered samples was determined using the free radical 2,2-diphenyl-1-picrylhydrazyl (DPPH) inhibition antioxidant assay, following a method based on those reported in literature^76,77^. A 0.5 mM DPPH radical solution in 100% methanol was prepared and kept in the dark for 2 h until the absorbance stabilized. After 2 h, 0.25 mL of the DPPH solution was added to the test tubes containing increasing amounts of pure or properly diluted phenolic extract in 0.5 mL of 70% methanol and 0.5 mL of 100 mM Na-acetate (pH 5.5). The solution was immediately mixed and left at RT in the dark for 30 min, after which the absorbance at 517 nm was measured. A mixed solution containing 0.5 mL 100% methanol and 0.5 mL 100 mM Na-acetate (pH 5.5) was used as a blank.

The antiradical activity was calculated as the inhibition ratio (%), which represents the amount of antioxidant required to reduce the initial DPPH concentration by 50% (IC50). This value was obtained using the following equation:

$${\text{Inhibition ratio }}\left( \% \right) = \left[ {\left( {{\text{Abs BLK}} - {\text{Abs sample}}} \right)/\left( {\text{Abs BLK}} \right)} \right] \times {1}00$$

The total antioxidant capacity was determined using a calibration curve of ascorbic acid (ASA) in a concentration range of 0–50 nM, similar to the approach adopted by^78^ to define its antioxidant kinetic behavior. A standard regression line (1) (y = ax + b) was generated by plotting increasing concentrations (x) of ascorbic acid (ASA) against their inhibition ratios (y). Two points that enclosed a 50% inhibition ratio were selected, and the obtained regression line (2) (Y = AX + B)^77^ was taken as a reference to calculate the sample concentration (X). The total antioxidant capacity was the average of the concentration values obtained for sample aliquots with inhibition ratios (Y) close to 50% and was expressed as µmol of ascorbic acid equivalents (ASAE) per gram DW.

### Isolation of structural biomass from corncobs and culture samples and thioglycolic acid lignin determination

Isolation of structural biomass (SBM) from powdered samples was conducted in triplicate, according to the method described by Brinkmann et al.^79^. Aliquots of 100 mg of powder were suspended in 10 mL of washing buffer containing 100 mM K_2_HPO_4_-KH_2_PO_4_ (pH 7.8), 0.5% (v/v) Triton X-100. Samples were gently stirred on a rotary shaker for 30 min at RT, then centrifuged at 5000 ×*g* for 20 min to recover the pellet. The pellet was resuspended in the washing buffer and treated as before to remove the organic fraction. The pellet was further washed four times for 30 min with 100% methanol (10 mL) to remove the lipid fraction. The final pellet, mainly consisting of cell walls (structural biomass, SBM), was oven dried at 70 °C overnight, weighted, and utilized for lignin analysis by the thioglycolate method, as described by Bruce and West^80^, with slight modifications^79,81^.

The lignin quantitative assay was conducted in triplicate by derivatization with thioglycolic acid (TGA) from powdered alcohol-insoluble sample residues. In screwcap 2 mL Eppendorf tubes, aliquots (10 mg) of SBM samples, as well as increasing aliquots (0–5 mg) of commercial lignin, were mixed with 1.5 mL of 2 N HCl and 0.15 mL of TGA. The screw caps were tightly closed, and the tubes were heated at 95 °C for 4 h to allow for the binding between TGA and lignin. The samples were then cooled in ice, centrifuged at 15,000 × *g* for 20 min at RT, and the pellets were washed three times with 1 mL of H_2_O. Subsequently, the pellets were extracted with 1 mL 1 N NaOH, in agitation for 16 h at RT. After extraction, the samples were centrifuged (15,000 ×*g* for 20 min at RT) and the resulting supernatants (1 mL) were recovered in 1.5 mL Eppendorf tubes. The supernatants were acidified with 0.2 mL HCl and incubated at 4 °C for 4 h to allow for the precipitation of the lignothioglycolate derivatives. The Eppendorf tubes were centrifuged (15,000 ×*g* for 20 min at 4 °C), the supernatant was discarded, and the final pellet was dissolved in 1 mL 1 N NaOH. After proper dilution, the total lignin content was measured at 280 nm. The data were expressed as mg of lignothioglycolate derivatives (TGAL)/g DW based on a comparison with a calibration curve generated by subjecting increasing amounts of commercial lignin, as a standard, to the same extraction procedure as the samples.

### Statistical analyses

The means and their standard deviations (SD) were calculated based on replicate values. All data were statistically analyzed using the two-way analysis of variance (ANOVA) (*P* < 0.05), along with the Tukey–Kramer test for multiple comparisons of the means. The statistical analyses were assessed using the statistical packages Prism GraphPad (version 6).

## Data Availability

All data are available in the text; any further details will be made available on request to manuela.rollini@unimi.it.
